# Arsenic and heavy metal contents in white rice samples from rainfed paddy fields in Yangon division, Myanmar—Natural background levels?

**DOI:** 10.1371/journal.pone.0283420

**Published:** 2023-03-24

**Authors:** Aye Myint Myat Soe, Aye Aye Mu, Kazuhiro Toyoda

**Affiliations:** 1 Graduate School of Environmental Science, Hokkaido University, Sapporo, Japan; 2 Department of Botany, Bago University, Bago, Myanmar; 3 Faculty of Environmental Earth Science, Hokkaido University, Sapporo, Japan; Universidade de Vigo, SPAIN

## Abstract

The presence of potentially toxic metal(loid)s (As, Pb, Cd, Cr, Mn, Fe, Zn, Cu, Ni, Mo and Co) in 120 white (polished) rice grains (*Oryza sativa*; 2017 or earlier crop) purchased from farmers in the five most agriculturally active townships near Yangon in the eastern edge on Ayeyarwady Delta was determined by triple quadrupole inductively coupled plasma mass spectrometry (ICP-QQQ). Their total-As and Ni concentrations (0.16 mg/kg, 0.39 mg/kg) were around the worldwide average literature values from a heavy metal non-contaminated area of intermediate to acidic (non-mafic) composition. Their Pb, Cd, and Cr mean concentrations (0.010, 0.0056, and 0.056 mg/kg, respectively) were lower than the maximum allowable levels by over one magnitude, reaching the concentration ranges comparable to the lowest level in the literature values. This study’s natural background levels were explained by a negligible influence of human, mining and industrial activities in this area, and probably genotype effect, which remains to be examined by the associated paddy soil analysis. Health risks associated with rice consumption (ca. 0.5 kg/day) by the inhabitants were estimated, assuming that inorganic arsenic was 30% of the total. Arsenic was the main contributor (30%) to the total value of the non-cancer risk (HI) of each element, which was 4.5 times the reference value (< 1), followed by Mn, Zn, Cu, Mo, Co and Ni (15–7%) and Pb, Cd, Cr and Fe (below 4%). The total cancer risk (TCR) for each element was around 17 times higher than the upper limit of cancer risk for an environmental carcinogen (< 0.0001): Nickel accounts for two-thirds of the contribution (66%), followed by Cd (16%) and As (13%). This study suggests that consumers of Yangon rice from paddy fields without groundwater irrigation may need to be concerned about the potential risk of Ni intake besides arsenic.

## Introduction

**Fig 1:**
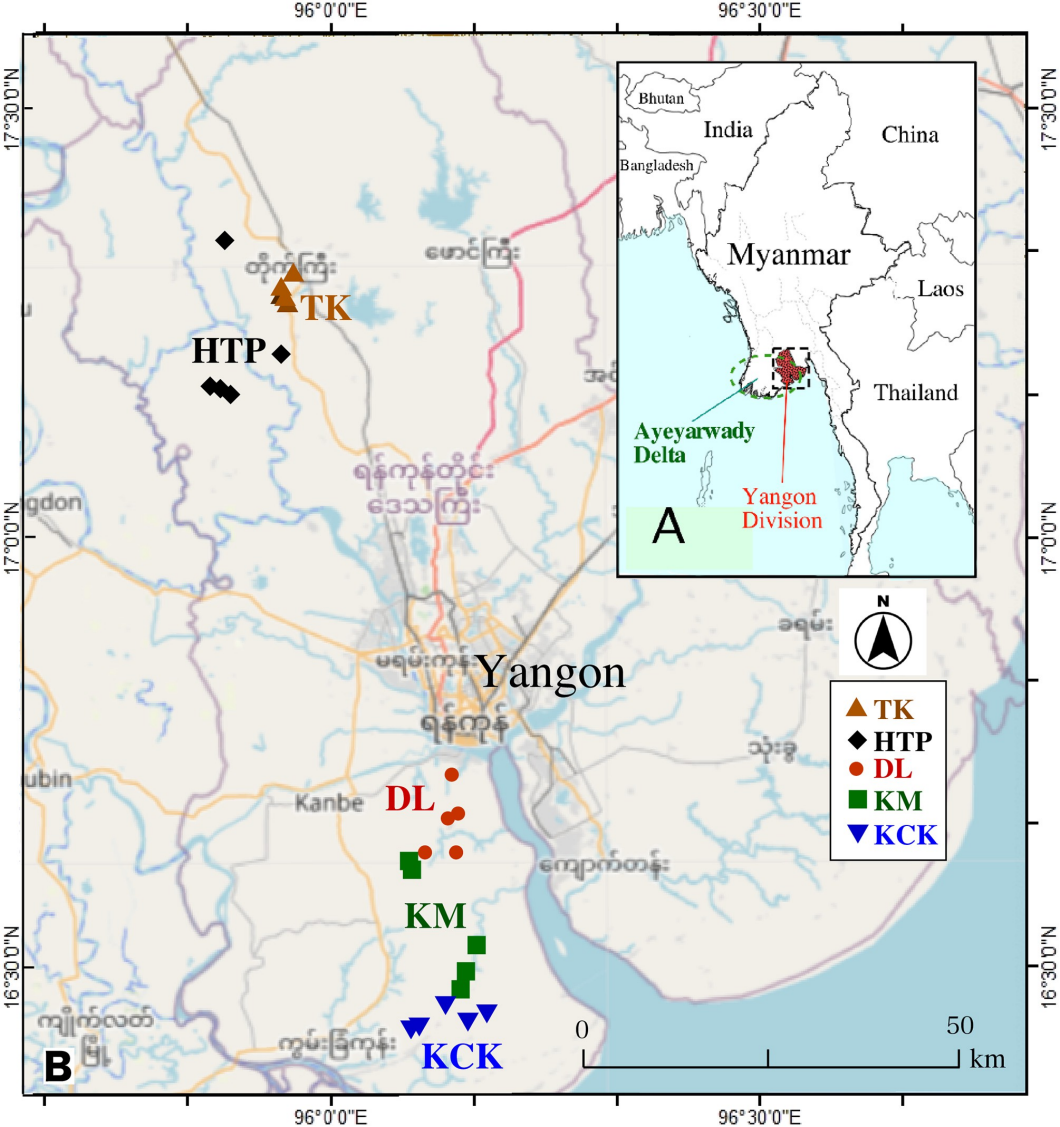
Maps of sampling areas in this study. A) Map of Myanmar and Yangon division (red full hatched area). B) The rice sampling points in five townships on the geographical map. The township names are as follows: Dala (DL), Taik Kyi (TK), Htan Ta Pin (HTP), Kaw Mhu (KM), and Kon Chan Kone (KCK). Figure made with 3Kaku-K company (https://www.freemap.jp/) and OpenStreetMap (https://www.openstreetmap.org/) sites.

Arsenic (As) is a well-known toxic element, and low-level arsenic poisoning symptoms include skin lesions, cardiovascular diseases, diabetes, and various organ cancers [1]. So, the cause of large-scale arsenic contamination in drinking water wells across Southeast Asia has been studied enormously since the mid-1990s (e.g., [2]). The natural arsenic release mechanism to groundwater is a microbial reduction of arsenic-bearing iron oxides in organic-rich sediment [3], mainly in the shallow reducing aquifer at the early stage of arsenic contamination of groundwater [2]. The poisoning areas’ characteristics are rapidly buried Holocene sediments with low hydraulic gradients in deltaic areas with rivers flowing from the Himalayas ranges [2], including Ayeyarwady (Irrawaddy) River Delta in Myanmar (Fig 1A). In the groundwater from below 20 m depth in the Ayeyarwady Delta area, elevated As concentrations (up to ca. 0.6 mg/L), higher than the WHO (World Health Organization) standard for drinking water (0.01 mg/L) [4], were reported [5, 6].

Rice (*Oryza sativa* L.) is the primary staple food in Southeast Asian countries. Years of paddy irrigation with the groundwater resulted in severe arsenic contamination in paddy soils and rice grains, inducing health concerns associated with rice consumption (e.g., [7]). Arsenic-accumulated rice samples are reported from these countries, e.g., 0.49 mg/kg of average As concentration in rice from Gazipur, Western Bangladesh [8]. Recently, more serious pollution in rice grains was reviewed [1, 9–11]. However, paddy irrigation with groundwater is quite rare in the Ayeyarwady Delta (20570 km^2^), which accounts for half of the total rice production in Myanmar [12]. The heavy monsoonal rains allow rainfed rice (*Oryza sativa* L.) fields with the usual continuous flooding irrigation in the Ayeyarwady Delta area, of which 60% is used for rice yield [13]. Therefore, the As concentration in the rainfed rice from the delta areas is expected to be a background level anterior to the groundwater irrigation. Recently, it was reported that inorganic arsenic contents in white rice grains from markets in the Ayeyarwady Delta area (including the Yangon deltaic region) were 0.04–0.07 mg/kg [14], below the maximum contamination level (Codex standards) (0.2 mg/kg) [15].

The accumulation of heavy metal contents in rice samples has also received tremendous attention to the health impacts in Asian countries and was comprehensively reviewed (e.g., [1, 11]). Cadmium (Cd) is a toxic element having a high risk of accumulating in rice grain as As (e.g., [16]), which is the cause of an epidemic occurrence of Itai-Itai disease (1910–1970, Japan) due to mining and smelter activities. The benchmark dose as the threshold limit level of Cd concentration in rice for Itai-Itai disease development was estimated as 0.27 mg/kg [17]. Cadmium is usually found in ores together with lead (Pb), copper (Cu), and Zinc (Zn). Nickel (Ni) is also a high-risk heavy metal accumulating in rice grain in the paddy soil derived from basalt rock [18], which is usually enriched in Ni, chromium (Cr) and cobalt (Co). These heavy metals (Cd, Pb, Cu, Zn, Ni, Cr, Co, Fe, and Mn) are widely used in industrial processes. The other symptoms of an overdose of heavy metals are Cd: damage to the kidney, liver, lungs, and the nervous system, cardiovascular diseases, development of reproductive toxicity, cancer, and mortality; Pb: renal dysfunction, damage to the central nervous system, and decrease of the intelligence quotients in children; Ni: genotoxicity, haematotoxicity, immunotoxicity, and carcinogenicity [1].

Although Yangon is the most industrialised city in Myanmar, little industrial activity has been found in paddy fields in the Ayeyarwady Delta area, far from any heavy-metal mining activity in Myanmar [19]. We can expect background levels of heavy metals and As in rice grain from the rainfed paddy field. However, we could find only a few data on heavy metals by ICP-OES analysis in the reports [20, 21]. So, we quantified As and these heavy metal contents in white (polished) rice samples (Yangon Rice, named in this paper) from the east end of Ayeyarwady Delta, five townships in Yangon Division (Fig 1A), Myanmar. The elemental content in white rice depends not only on the concentration in the soil but also on the growing conditions and the genotype of rice [22]. Assuming that the growing conditions and rice varieties in the region will remain the same in the future, it is essential to know the concentration of harmful elements before human activities to identify artificial contamination. By comparing the literature values worldwide, we will highlight the low level of heavy metal elements in rice in this study and consider the accumulation factors from previous reports. This study tried to translate the data’s variation, assuming that rice grains’ element levels depend on the elemental abundance and mobility in the associated paddy soil, assessing the potential health risk (non-cancer and cancer risks) of each element for the rice consumers. This study aims to confirm Yangon rice’s safety and contribute to the food safety sustainability and awareness associated with rice consumption.

## Materials and methods

### Sampling sites

Myanmar is the country in the hatched area in Fig 1A. The high-altitude northern part of Myanmar connects to the Tibetan Plateau, while the southern part has a low altitude and faces the sea. The Ayeyarwady River flows in the sedimentary basin’s central zone and forms the Ayeyarwady Delta by the Quaternary sediment at the southern end. Some Cu-Au mines distribute in the northern half of the central zone, and tertiary intermediate to acidic volcanic rocks and Cenozoic limestones scatter the zone’s centre.

A total of 120 rice samples were collected from farmer houses in five different townships in April 2018. The five townships, 25 sampling sites, are located in the Yangon division, an eastern part of the Ayeyarwady Delta (Fig 1B). This location is sometimes called the “Yangon deltic region” for administrative reasons. However, this paper calls this area part of the Ayeyarwady Delta from a topographical view. Since these five townships are the most agriculturally active areas and have no non-farm dwellings, the samples in this study can be regarded as the least affected by human activities. The latitude and longitude data of the sampling point were obtained with the GPS tool, and the altitude data were by Google earth software. The altitudes of the northern two townships, TK (Taik Kyi) and HTP (Htan Ta Pin), are almost 11–18 m on the upstream side; The altitude of the southern three townships, DL (Dala), KM (Kaw Mhu), and KCK (Kon Chan Kone), is almost in 4–9 m on the downstream side (S1 Table).

Tropical cyclonic storms generate heavy rainfall and severe flooding in Myanmar between May and October [13]. Because the study area is a lower floodplain area, rice paddies in this region remain constantly flooded from sowing to earning. Rice harvesting is usually done after the water naturally recedes at the end of the rainy season. Observations of redox potential changes in other countries have reported that paddy soils above 10 cm water depth have continuously maintained a reduced state (approximately -200 mV to -300 mV vs. SCE) (e.g., [23]). The SCE (saturated calomel electrode) is a reference electrode based on the reaction between elemental mercury and mercury(I) chloride. It can be assumed that the paddy soil of the rice samples analyzed in this study would have also maintained a continuously reducing state during the rice growing. In these areas, the irrigation water source is the rainwater on the ground, including river water, but farm pond water is also available only in TK township. Rice production is carried out only during the rainy season (May–October). No metal mines are found within 100 km of the sampling sites [19].

### Analytical procedure

All 120 samples were treated on a clean bench, digested with certified reference materials (CRMs) and blank samples in runs. About 10 g of collected white (polished) rice grains were finely powdered by agate mortar, homogenised, and dried in a hot air oven (55°C for 36 h) until the weight became constant. However, we follow the direction for the use of four certified reference materials (NMIJ CRM 7501-a, 7502-a; NIES CRM No.9, 10; S2 Table) on the weight basis after drying at 85°C for 4 hours. The moisture loss was 6–7%. Each powdered dry sample (30 mg) was weighed and placed in a 7 ml PFA vial (Savillex) and then mixed with 1mL of ultrapure-grade 69% (v/v) nitric acid and 0.1 mL of 30% (v/v) H_2_O_2_ (Kanto Chemical). After the digestion in the closed PFA vial in a hot air oven at 80°C for 12 hours, each sample solution was diluted into 30 mL with ultrapure water (milli-Q, Simplicity UV and Gradient with Elix10, Millipore) from the cleanroom facility of the school. Only Sargasso CRM was digested by the microwave digestion system (Ethos One, Milestone General). The digestion program was created at the 25-minute ramp and 5-minute hold at 180°C.

Eleven elements in all sample solutions stocked in clean polypropylene bottles were determined with an Agilent 8800 triple quadrupole ICP-MS (ICP-QQQ) at the Open Facility of Hokkaido University. Arsenic was determined by using mass-shift mode for As^+^ from m/z 75 to AsO^+^ at m/z 91 to eliminate the mass interference by ^150^Nd^2+^ and ^150^Sm^2+^ [24]; and the other elements (Pb, Cd, Cr, Mn, Fe, Zn, Cu, Ni, Mo, and Co) were with helium collision mode. The sweep times were 50–100, and integration times were 3.0 x 3 sec for As, Pb, Cd, Cr, and Co; 0.1 x 3 sec for Mn and Fe; 0.3 x 3 sec for Zn, Cu, Ni and Mo. All working and running standard solutions (0, 5, 20, 50, and 100 μg/L) were prepared by serial diluting ICP multi-element standard solution H (Kanto Chemical) with ultrapure water. Data precision and accuracy were checked by analysis of four certified reference materials, and no systematic error was observed (S2 Table). The limitation of quantitation (LOQ = standard deviation (SD) ×10) was calculated from the measurement of different blank samples and converted into rice sample abundance when 30 mg of a sample was digested into a 30 mL solution. It is estimated that analytical error ranges between 5–15% for the measurement of LOQ concentration; 2–4% for one order of magnitude higher than the LOQ concentration.

### Risk calculations

The health risk assessment method is established by the Joint FAO (Food and Agriculture Organization)/WHO (World Health Organization) (e.g., [25]) and Expert Committee on Food Additives and the United States Environmental Protection Agency (USEPA) (e.g., [26]). The average daily intake (mg/kg/day) of heavy metals through ingestion of rice grains can be estimated as follows:

EDI(Estimateddailyintake)=Cm×IR/BW (1)

where Cm (mg/kg): mean heavy metal concentration in grains; IR (g/day): ingestion rate of rice; BW (kg): average body weight. Incidentally, the daily rice consumption in Myanmar was reported to be 578 g/day before 2000, 512 g/day in 2014, and 487 g/day in 2017 [https://www.helgilibrary.com › indicators › myanmar]; we set IR at 500 g/day as the convenient value to consider in this study. We put 58 kg of average body weight into BW since the mean body weights between 18 and 25 years of age are 61.1 kg for males and 54.7 kg for females in Myanmar [https://www.worlddata.info/average-bodyheight.php].

The hazard index (HI) of non-carcinogenic risk by rice consumption is estimated as the sum of each toxic element’s non-carcinogenic hazard quotient (HQ) values. So, the HI (hazard index) is equivalent to the total HQ (THQ). The HQ values are calculated as follows:

HI=THQ=ΣHQ=Σ(EDI/RfD) (2)

where RfD (mg/kg/day) is the oral reference daily dose of each heavy metal. Chronic oral RfDs should be used to evaluate the potential non-carcinogenic effects associated with exposure periods greater than seven years [26]. “HI (THQ) > 1” indicates that the level of exposure is likely to cause non-carcinogenic health risks to an individual, whereas chronic risks are assumed to unlikely happen in case HI < 1 [27].

Total cancer risk (TCR) is the sum of cancer risk (CR) values for each carcinogenic metal(loid) (As, Cd, Cr, Ni and Pb). The CR was calculated by multiplying the EDI (mg/kg/day) over a lifetime with a slope factor (SF) for an incremental risk of individual developing cancer, using the equation provided in USEPA Region III Risk-Based Concentration Table [28]:

$$\text{TCR}=\Sigma \text{CR}=\Sigma (\text{EDI}\times \text{SF}\times 0.371)=\Sigma \left\{\text{EDI}\times \text{SF}\times (\text{EF}\times \text{ED}/\text{AT})\right\}$$ (3)

where EF is the exposure frequency (365 days/year), ED is the exposure duration (26 years) [26, 28], and AT is the averaging time for carcinogens (365 days/year × 70 years). The child exposure duration is limited to 6 years, and the adult exposure is assumed to be the entire 26 years [26], so the results of this study are for middle-aged and older adults. The TCR indicates the cumulative probability of an individual developing cancer over time. For example, a TCR of 10^−4^ indicates a probability of one person developing cancer in 10,000 individuals. USEPA designated that the TCR value between 10^−6^ (threshold level) and 10^−4^ (residual level) are acceptable risks for causing cancer [26]. Incidentally, the life expectancy of Myanmar males is 64 years, and that of females is 70 years [https://www.worlddata.info/asia/burma/index.php].

The non-carcinogenic hazard quotient (HQ) values of each toxic element from Yangon rice consumption were calculated by Eq (2) using RfD (Oral Reference Dose) values of 0.0003, 0.003, 0.001, 0.003, 0.14, 0.7, 0.3, 0.04, 0.011, 0.005, and 0.0003 for inorganic As, Pb (phosphate), Cd, Cr(VI), Mn, Fe, Zn, Cu, Ni (hydroxide), Mo and Co, respectively [29, 30]. The lifetime cancer risk, the probability of an individual exposed to a specific carcinogenic metal(loid) (As, Pb, Cd, Cr and Ni) developing cancer during the lifetime, was also calculated by Eq (3) using oral slope factors (SFs) values of 1.5, 0.038, 15.0, 0.50, and 0.91 for inorganic As, Pb (phosphate), Cd, Cr(VI), and Ni (hydroxide), respectively [18, 30].

## Results and discussion

### Elemental abundance in white rice grains

**Fig 2:**
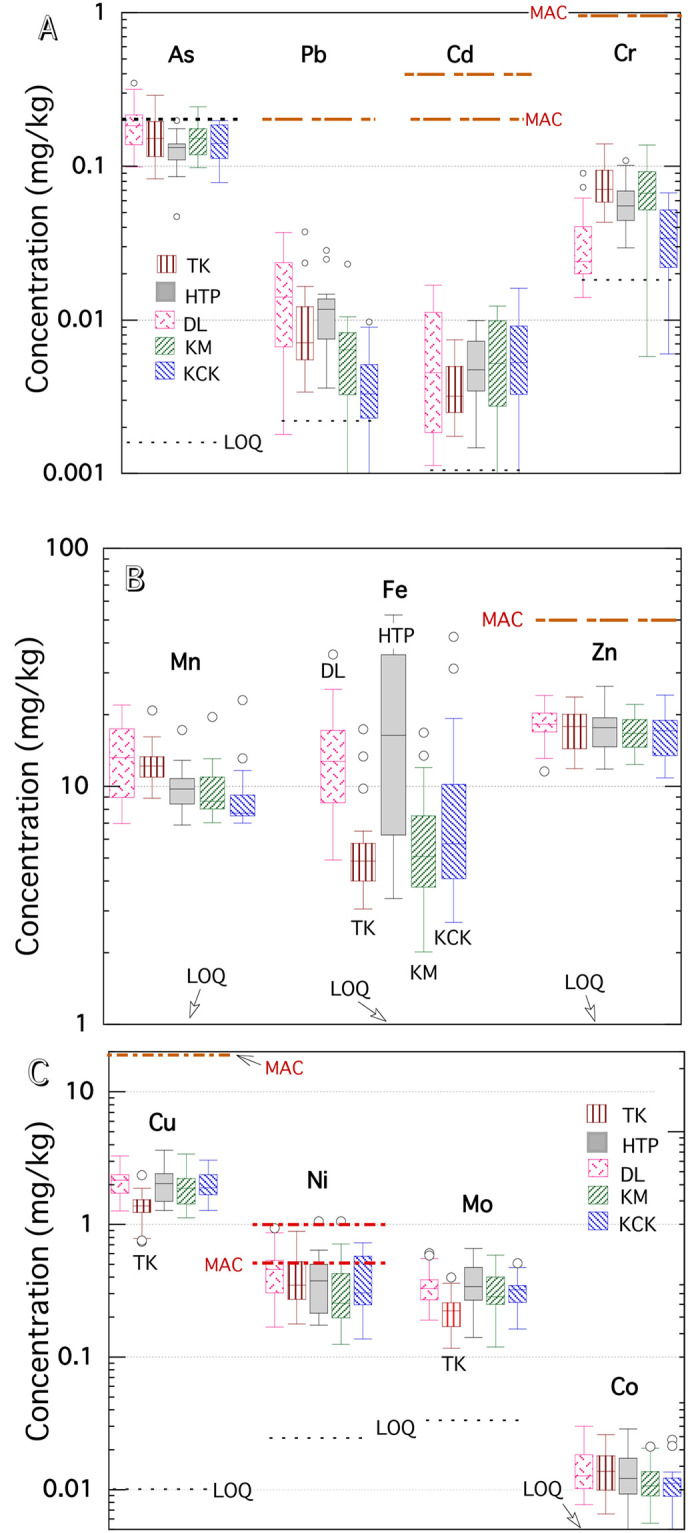
Box plot of each element concentration in rice samples from five townships. A) As, Pb, Cd, and Cr; B) Mn, Fe and Zn; C) Cu, Ni, Mo and Co. The horizontal line in a shaded box encloses 50% of the data, indicating the median value. The box’s top and bottom edges (UQ and LQ) mark 75 and 25 percentiles, and the difference between UQ and LQ is the interquartile distance (IQD). The lines extending from each box’s edges indicate the maximum and minimum values between "UQ + 1.5 x IQD" and "LQ—1.5 x IQD" values. Any outlier value outside the range is displaced as an individual point. The dash-dot and dotted horizontal lines at each element are the maximum allowable concentrations (MAC) in white rice grain [15, 31] and the limits of detection (LOD) of the analysis of this study, respectively (Table 1).

Fig 2A–2C demonstrate the concentration distribution of 11 elements (As, Pb, Cd, Cr, Mn, Fe, Zn, Cu, Ni, Mo, and Co) in white rice grains by five sampling townships (TK, HTP, DL, KM and KCK; in order from north to south in the map of Fig 1B). Most of the data are above the limit of quantitation (LOQ; dotted line in Fig 2A–2C) in the analysis: Only a few or dozen Pb, Cd and Cr analysis data were below LOQ (S3 Table). Table 1 shows the mean concentration of 11 elements in 120 samples of this study, LOQ of this analysis, and maximum allowable concentration (MAC) values (dot and dash lines in Fig 2A–2C) of 8 elements (iAs (inorganic As), Pb, Cd, Cr, Zn, Cu, and Ni) [15, 31] from the FAO/WHO and USEPA guidelines. The mean and medium values of Pb, Cd, Cr, and Cu in 120 white rice grains were one order of magnitude lower than the corresponding MAC values; The upper quartile values of As and Ni contents in our samples were equivalent to the MAC values.

**Table 1:** Elemental concentration data in white rice samples, estimated average dietary intakes (EDI), and the potential health risks (CR per 10,000 people and HQ) with mean and standard deviation (SD) values.

Yangon white rice	**As**	**Pb**	**Cd**	**Cr**	**Ni**	
**Level (μg/kg)**	155 ± 52	9.7 ± 8.2	5.6 ± 3.7	56 ± 29	393 ± 192	
**Range (μg/kg)**	47–348	(0.7)–37.4	(0.3)–16.8	(6)–140	120–1060	
**LOQ**^b^ **(μg/kg)**	1.6	2.2	1.0	18	25	
**MCA** ^c^ **(mg/kg)**	0.2 (iAs)	0.2	0.2/0.4*	1.0	0.5*/1.0	
**EDI** ^d^ **(μg/kg-day)**	1.34 ± 0.45	0.084 ± 0.070	0.048 ± 0.032	0.49 ± 0.25	3.39 ± 1.65	
**TDI**^d^ **(RDA**^d^**)**	2.1 (iAs)	3.6	1	3.33	5.0	
**RfD** ^**e/**^ ^f^ **(mg/kg-day)**	0.0003 (iAs)	0.003	0.001	0.003	0.02/0.011	
**HQ** ^f^	4.46 ± 1.51	0.028 ± 0.023	0.048 ± 0.032	0.16 ± 0.08	0.31 ± 0.15	
**SF** ^c^ **/SFO** ^g^ **(1/mg/kg-day)**	1.5 (iAs)	0.0085	15.0	0.50	1.7/0.91	
**CR**^f^ **(per 10,000 people)**	7.5 ± 2.5	0.012 ± 0.010	2.7 ± 1.8	0.90 ± 0.47	11.4 ± 5.6	
Yangon white rice	**Mn**	**Fe**	**Zn**	**Cu**	**Mo**	**Co**
**Level (mg/kg)**	10.9 ± 3.7	11.9 ± 14.9	17.3 ± 3.3	1.90 ± 0.60	0.32 ± 0.11	0.013 ± 0.005
**Range (mg/kg)**	6.9–23.0	2.0–52.6	10.9–26.3	0.74–3.64	0.12–0.66	0.004–0.030
**LOQ**^b^ **(mg/kg)**	0.013	0.625	0.072	0.0095	0.033	0.0025
**MCA** ^c^ **(mg/kg)**			50	20		
**EDI** ^d^ **(μg/kg-day)**	94.1 ± 31.5	103 ± 129	149 ± 29	16.3 ± 5.1	2.73 ± 0.99	0.112 ± 0.046
**TDI**^d^ **(RDA**^d^**)**			1000	71.4		
**RfD** ^**e/**^ ^f^ **(mg/kg-day)**	0.14	0.7	0.3	0.04	0.0050	0.00030
**HQ** ^f^	0.67 ± 0.23	0.15 ± 0.18	0.50 ± 0.10	0.41 ± 0.13	0.55 ± 0.20	0.37 ± 0.15

^a^: Results below LOD (limitation of detection) are shown as the LOD value in parentheses.

^b^: Limitations of quantitation (= standard deviation (sd.) x 10) of blank solutions in measurements were converted into rice sample abundance (mg/kg).

^c^: MAC: Maximum allowable concentration ([15]; *[31]); bw: body weight.

^d^: EDI: Estimated daily intake; TDI: Tolerable daily intake; RDA: Recommended daily allowance [9, 10]

^e^: RfD (Oral Reference Dose); SF (Slope factor); HI: Hazard Index; TCR: Total CRs

^e^: RfD and SF values were compiled by [29, 32].

^f^: Non-carcinogenic and carcinogenic risks for As and Cr were calculated based on 100% inorganic As and Cr(VI) in rice samples.

^g^: Regional Screening level (RSL) Summary Table 2022 May (TR = 1E–06, THQ = 1.0) [30]

Table 1 indicates that the total-As content in 120 samples was 0.155 ± 0.052 mg/kg, and the FAO/WHO proposed MAC of inorganic As (iAs) at 0.2 mg/kg for white rice [15, 33]. The iAs concentration in Yangon rice grains was not measured in this study. The iAs is considered fairly more toxic than methylated As species [34] and is a non-threshold Class 1 carcinogen. It was reviewed that 0.08–0.20 mg/kg was a normal global range for As concentration in rice; 0.14–0.16 mg/kg was the global average value for long-diameter white-colour rice [35, 36]. Most of the As concentrations in this study are close to the average rice concentration globally. Table 1 also indicates the tolerable daily intake (TDI) or recommended daily allowance (RDA) of inorganic As and some other elements (μg/kg body weight (bw)/day): TDI (= RDA) of inorganic As and Ni are 2.1 (μg/kg bw-day) and 5.0 (μg/kg bw-day) [9, 10]. On the contrary, the EDI of As and Ni in Yangon rice were 1.34 ± 0.45 and 3.39 ± 1.65 (μg/kg bw-day). For Pb, Cd, Cr, Zn, and Cu, the EDI values by Yangon rice consumption were smaller than the TDI values by about an order of magnitude. It is consistent with Fig 2A and 2C that arsenic and nickel intake might exceed the permissible range for the habitants.

Fig 2B demonstrates that Fe levels in rice grains from the HTP and DL townships are significantly higher than those in the other three townships (HTP>KCK: p = 0.009; DL>KCK: p = 0.046). It may translate that paddy soil in the HTP and DL townships is more enriched in the amorphous iron hydroxide phase (e.g., ferrihydrite) than in the other three townships. Iron is primarily mobilised chiefly as dissolved Fe(II) under reducing conditions, and it is redistributed as particulate Fe(III) oxyhydroxides in oxygenated environments [37].

Fig 2C shows that Cu and Mo levels in TK rice grains are significantly lower than in the other four townships (TK<KM and the other three; Cu: p = 0.0005; Mo: p = 0.0011). Of the five townships, only TK paddy fields used irrigation water from agricultural ponds, so rice’s low Cu and Mo levels may be due to differences in irrigation water sources. However, it is required to study rice and associated soil samples from several different townships. Determining phosphorus concentration should also be involved, as much remains to be clarified about whether adding sufficient phosphorus concentrations to the soil can reduce As accumulation in rice grains (e.g., [38]).

### Elemental correlations with arsenic contents

**Fig 3:**
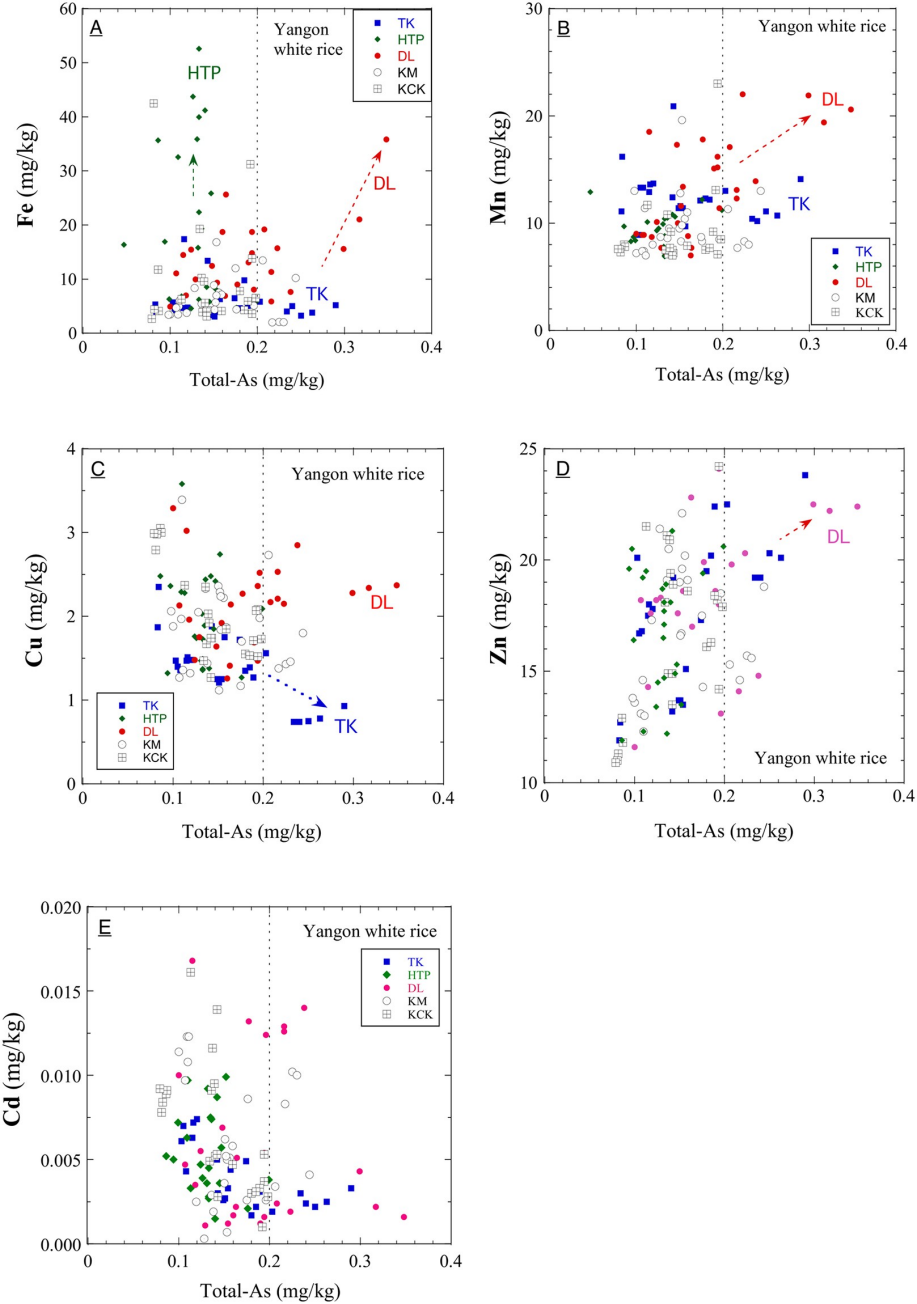
Elemental correlation with total-As contents in white rice grain. A) Fe; B) Mn; C) Cu; D) Zn; E) Cd concentrations in the grains of this study.

Fig 3A–3E show the correlation between As content and Fe, Mn, Cu, Zn, and Cd in this study’s grains. Fig 3A suggests that the upstream HTP and downstream DL townships’ paddy soils contain non-As-bearing and As-bearing iron oxide, respectively. Fig 3B implies that the paddy soils from DL have high Mn contents. It is well known that arsenic-polluted groundwater usually has a high Mn and Fe concentration as a worldwide phenomenon (e.g., [39]). For example, it was reported that a large amount of arsenic released from Holocene sediments, derived from the Himalaya ranges, to groundwater at a shallow valley was associated with a more significant elevation of manganese (Mn) levels in Cambodia [40]. Among the five sampling sites, the altitudes of the upstream sides (TK, HTP) and downstream sides (DL, KM, and KCK) are average about 12 m and 6 m, respectively (S1 Table). Google Earth shows that to the west of the downstream sites (DL, KM, KCK) area of about 6 m in height, there is an extended plateau ranging from 10 m to 25 m. The northmost DL site among downstream sides may be where arsenic-rich sedimentary layers eroded upstream could be carried and quickly deposited topographically during floods (Fig 1). Therefore, the migration of Himalaya-derived As-bearing iron oxide particles may explain the high As content in some DL rice grain samples. This hypothesis needs to be confirmed by soil analysis.

Fig 3C shows that the low Cu contents in TK rice (Fig 2C) might be associated with the high As contents likewise Fe contents in the rice. It was reported that the suppression effect of Cu on As accumulation in white rice [41]. Iron oxide plaque around rice roots is a strong adsorbent for As (V) and acts as a barrier to As uptake into the root cells [42]. It is plausible that the top-layer pond water for irrigation is depleted in bio-essential elements, Fe, Cu and Mo, relative to flood water containing suspended matter more abundantly because these elements easily incorporate sinking particles, including insoluble hydroxides and microorganisms in water. Therefore, we can infer that pond water irrigation contributes to the Cu and Fe-depleted paddy soils and high As contents in rice grains at TK township. However, it remains to be confirmed and clarified by the soil and water analysis in future.

Fig 3D shows a positive correlation (r = 0.47) between Zn and As in the white grains in this study. Previous studies have also shown, for example, positive correlations of Zn-As (r = 0.24) and of Cu-Zn-Cd in rice from an arsenic-unpolluted area (rice grain As 0.11 ± 0.04 mg/kg) in China were demonstrated [43]. The heavy metal concentrations explain these positive correlations in soils, usually depending on Mn oxides because manganese oxides adsorb these heavy metals with high capacities [44] and iron oxides [45].

The redox potential of paddy soil significantly affects the bioavailability of As and Cd, and it has profound opposite effects on the concentration of As and Cd in rice grains [23]. This study expected a negative correlation between As and Cd in white rice grains, and a weak negative correlation (r = –0.27) was found across all five regions. Also, significant negative correlations in township samples except DL were found (TK: –0.54; HTP: –0.60; KCK: –0.63; KM: –0.31; DL: –0.14; S4 Table). The non-significant negative correlation for DL township may be related to the significant positive correlation between iron and arsenic in samples only from DL township (DL: 0.55; TK: –0.18; HTP: 0.00; KCK: –0.09; KM: –0.13; S4 Table). The presence of arsenic-containing iron components in the soil only in DL areas may influence the behaviour of arsenic in the soil more strongly than pH or redox potential in paddy soil. This hypothesis needs to be confirmed by chemical analysis of the soil soon.

Generally, there are significant differences in the bioaccumulation of toxic elements in rice grains by genotype [22]. However, in the next section, we will compare the four elements by country/region based on literature values without distinguishing differences by genotype.

### Cadmium contents in rice grains from the world

**Fig 4:**
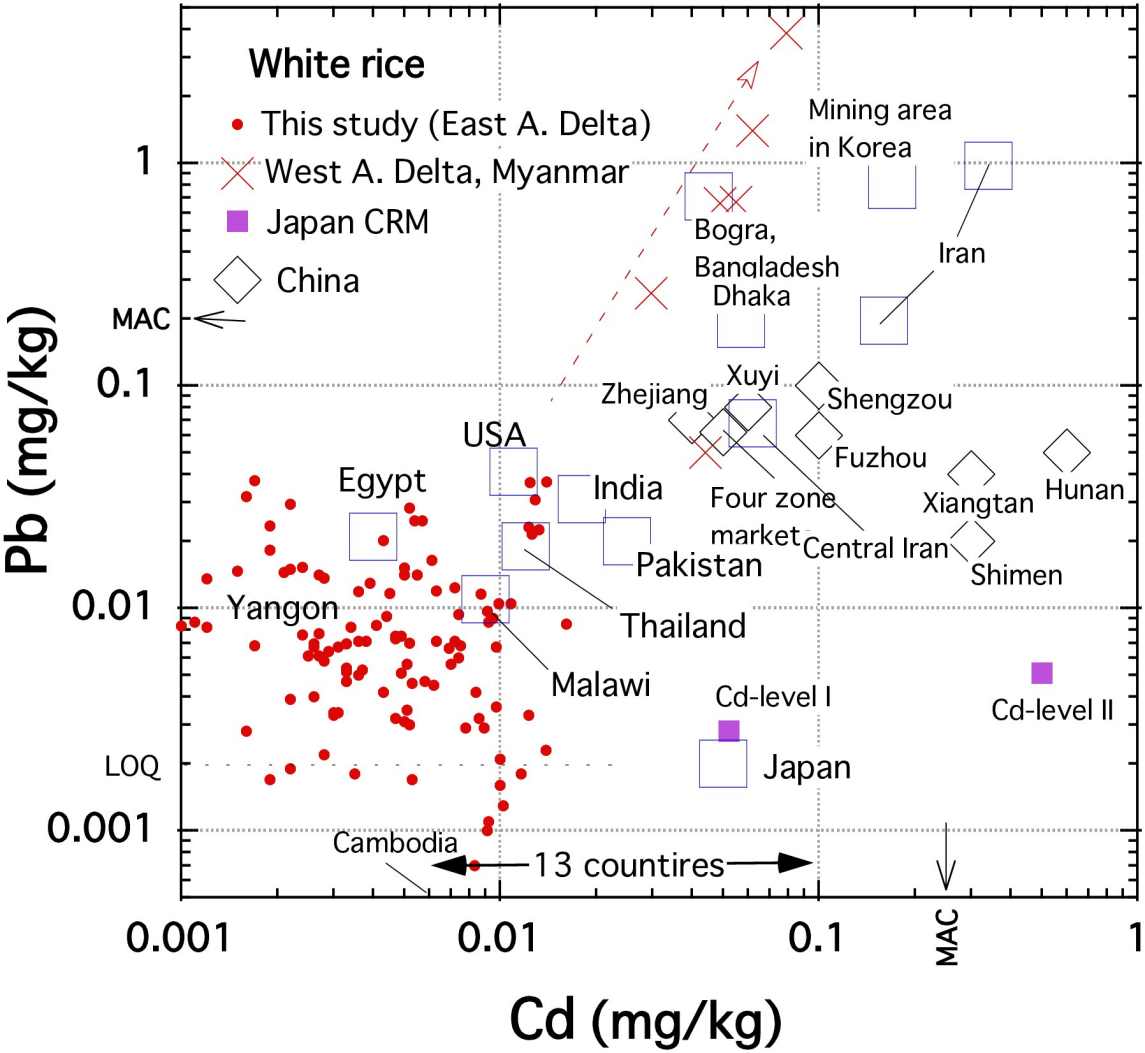
Correlation diagram between Pb and Cd concentrations in white rice from different countries and areas. Japanese certified reference materials (CRM) data are from S2 Table. The mean values of previous reports: West Ayeyawady delta in Myanmar [20, 21]; Xuyi, China [18]; Shengzou, Shimen, Fenghuang, and Xiangtan, in China [47, 48]; Bangladesh [49, 50]; Iran [51, 52]. The Cd range of 13 countries (Cambodia, France, Ghana, Italy, India, Japan, Nepal, Spain, SriLanka, Thailand, USA, Bangladesh, Jamaica) [45, 53]; China [54–57]; Japan [58]; Korea [59]; Malawi [46]; Central Iran [60]; Egypt, India, Pakistan, Thailand and USA [7].

Fig 4 is a scatter plot of Cd-Pb concentrations in this study compared to the reported mean concentrations in white rice grains from various countries and regions (The data sources are described in the figure caption). In this study, the Cd concentrations in white rice grains were 5.6 ± 3.7 μg/kg (n = 115; Table 1). It is comparable to the lowest concentration in the literature values (Fig 4). Previous papers reported that the white rice from Cambodia has the lowest mean Cd contents (6 μg/kg: n = 14) among the ones from 12 countries [45]; Egypt rice has 4 ± 1 μg/kg (n = 5), the lowest concentration among eight countries [7]; the mean Cd concentration in white rice from four areas in Thailand and rice from Saraburi provinces has 6 ± 4 μg/kg (n = 5) Cd concentration [10]; Malawian rice’s mean Cd content is 9 μg/kg (n = 21) [46]. Therefore, Yangon rice is one of the rice grains with the lowest Cd content globally.

The Cd concentrations in rice grain vary by three orders of magnitude, depending on rice genotype and growing conditions (pH and redox condition) associated with the bioavailability of Cd in soil [16]. The median Cd transfer ratio from soil to grain increases by two and a half orders of magnitude when the soil pH value drops from 8 to 5 [61]. Volcanic acidic soil is usual in Japan, a highly volcanic country with 110 active volcanoes. It can be one of the reasons that the Cd average content in white rice collected in 1998–2000 in Japan is 50 μg/kg (n = 1198; 65 sites; [58]), an order of magnitude higher than the ones in samples of this study (Fig 4). In contrast, Thailand’s Saraburi province (rice Cd contents: 6 ± 4 μg/kg) is a sedimentary carbonate area. The paddy soils of Yangon rice may not be calcareous. However, they are certainly not acidic due to Myanmar’s geology: no volcanic activity and several limestone mines are found in the middle stream of the Ayeyawady River [62].

The redox potential of flooded paddy soil is usually held in reducing conditions (–200 mV vs SCE; [22]). Although the soil pH was slightly acid to neutral (6.4 ±|0.8) in the study area [63], the seasonal changes in surface water pH during the rice-growing season were in the range of 7.0–8.0 in the upstream area [64]. The Eh-pH diagram of Cd [65] indicates that most Cd exists in the form of CdS, which has low solubility around neutral pH (log Ksp = –14.4; [66]). Since the study area in the Ayeyawady delta is annually exposed to flooding during the heavy monsoonal rainy season, the soil is under reducing conditions. The irrigation system should contribute to the rice grains’ low Cd concentration.

### Lead contents in rice grains from the world

This study showed that the Pb mean concentration in white rice grains was 10 μg/kg (n = 115; Table 1). It corresponds to the second-lowest concentration after Japan (1.8–3.0 μg/kg; n = 1198; 65 sites; [58]). It was reported that the mean Pb content of Malawian rice was 11 μg/kg (n = 21) [46]; 17–20 μg/kg (n = 4–22) was the Pb median concentration in rice grains from Thailand, Pakistan, India and Egypt [7]. So, Yangon rice is one of the rice samples with the lowest Pb contents (Fig 4).

The lead (Pb) has a lower solubility than cadmium in the paddy soil of the delta. The Eh-pH diagram of Pb [65] suggests that most Pb exist in the form of PbS or PbCO_3_, which have low solubilities (log Ksp = –27.9, –13.1, respectively; [67]) in the condition (pH = around 7; –200 mV vs SCE; [22]). The hydrous ferric oxide, formed at the soil surface under the floodwater, is known to have excellent adsorption properties against Pb ions and other toxic ions (e.g., [68]). Investment of phosphate-based fertiliser into paddy soils promotes the formation of stable lead phosphate mineral pyromorphite [Pb_5_(PO_4_)_3_(OH ·Cl)] [69], which has very low solubility (log Ksp = –79) [70]. Myanmar’s agricultural policy (http://www.fao.org/3/ca3662en/ca3662en.pdf) recommends fertilisation (commonly, P 30 kg/ha) [64]. The associated high phosphate levels in paddy soils may be one of the reasons for the low Pb levels in Yangon rice. The extractable P level in nearby paddy fields was 0.3–195.7 mg/kg [77].

One of the reasons for the low Pb content of Yangon rice might be the wind direction during the rice growth period. This study’s two sampling sites are 2 km north-northwest of Yangon urban, and the other three are within 2 km just south of the area. Southwest wind prevails during this area’s rainy season (May–October) (https://weatherspark.com/y/112503/Average-Weather-in-Yangon-Myanmar-(Burma)-Year-Round). Hence, we assumed that rice plants in the sites grew without exposure to the Yangon urban area’s aeolian dust, 5.2% of roadway emissions [71]. Many countries have reported high Pb contents in roadside soils [72]. Therefore, the weather may allow this study’s grain samples to have little traffic-related lead pollution, although the sites are near the metropolitan.

### Nickel and chromium contents in rice grains from the world

**Fig 5:**
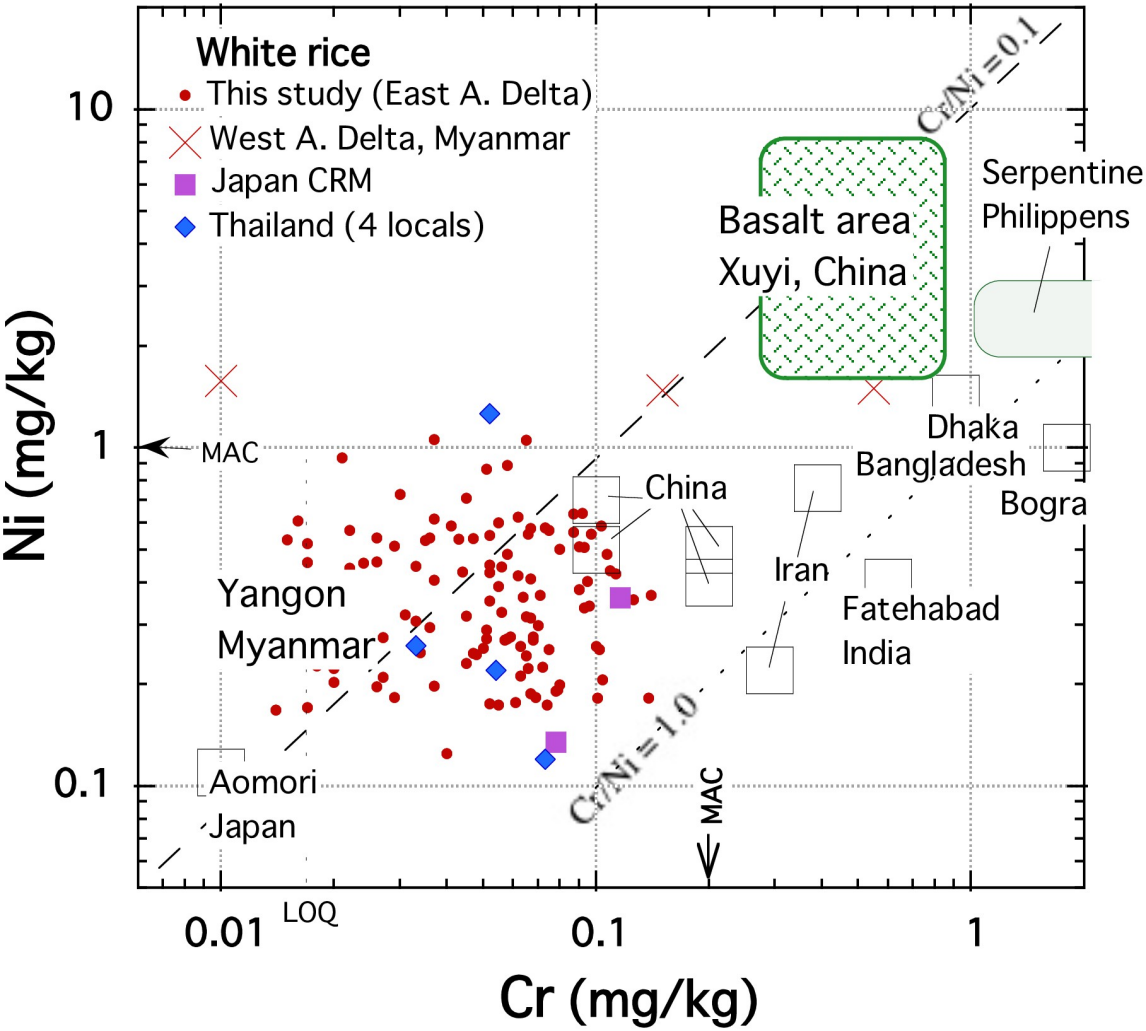
Correlation diagram between Ni and Cr concentrations in white rice from different countries and areas. Japan certified reference materials (CRM) data are from S2 Table. The mean values or the level ranges of previous reports: West Ayeyawady delta in Myanmar [20, 21]; Japan certified reference materials (NMIJ CRM 7501-a and 7502-a; S2 Table) Xuyi, China [18]; Shengzhou, Shimen, Fenghuang, and Xiangtan, in China [47, 48]; Bangladesh [49, 50]; Iran [51, 52]; Four local areas in Thailand [10]; Zambales, Philippines [73]; Fatehabad, India [74]; Aomori, Japan [75]. Incidentally, Aomori has one of the lowest population densities in Japan.

Fig 5 shows the Ni-Cr plot of concentrations of previous reports and this study. It is plausible that the associated paddy soil concentrations primarily determine the Ni and Cr contents in rice grains. In contrast to the MAC values in white rice (1.0 mg/kg of Ni and 0.2 mg/kg of Cr), 2–3 mg/kg of Ni and 1–6 mg/kg of Cr contents are found in the rice grain grown on serpentine soils, the weathered product of peridotite host rock (ultramafic rock) in Philippines (a small patterned square in Fig 5) [73]. The rice grown on the basaltic soil in areas of low human activity in Xuyi County, China, has 1.22–9.34 mg/kg of Ni and 0.24–0.91 mg/kg of Cr as the range of contents (a sizeable patterned rectangle with rounded corners in Fig 5) [18]. The Ni, Cr and Co concentrations in Yangon rice grains were 0.39 ± 0.19 mg/kg, 0.056 ± 0.029 mg/kg and 0.013 ± 0.005 mg/kg (n = 118–119; Table 1), which are as low as the literature values of white rice from Japan and Thailand, where the distribution area of mafic rock is few (https://ccop-gsi.org/main). Felsic rocks distribute widely in Japan along the island arc volcanic zone. The low Ni and Cr content of Yangon rice is presumably due to the low concentration of Ni and Cr in the paddy soils, as there are no mafic rocks in the vicinity of the sampling points. However, the content of the associated soil remains to be examined in future.

Fig 5 suggests that white rice samples from two Chinese locations, Iran, India, and Bangladesh [47–52], project towards a Cr enrichment component, which is different from the trend of the dashed line (felsic–mafic rock area products: Cr/Ni = ca. 0.1) by the rock type difference connecting literature values for samples from areas with low human activity.

These paddy fields (Xiangtan, Shengzhou, Fatehabad, Dhaka, and Bogra) are out of basaltic rock areas (https://ccop-gsi.org/main). The Cr enrichment in rice might be due to Cr pollution by industrial activities such as metal plating, wood preservation, ink manufacture, dyes, pigments, glass and ceramics, tanning and textile industries, and corrosion inhibitors in cooling water [76].

On the other hand, industrial Ni soil contamination seems insignificant compared to mining and refining nickel mines activities. However, our daily lives are full of nickel alloy parts, and nickel intake can cause various effects on human health [77]. The livelihoods of farmers who cultivate this paddy field area may be affected. The correlation tables between ten elemental abundances in sample grains by townships (S4 Table) reveal that Ni contents have high positive correlations with both Cu and Cd contents (Cu: r = 0.70–0.88; Cd: r = 0.59–0.73, except KM township). Slightly higher correlation coefficients (Cu: r = 0.83, 0.84; Cd: r = 0.73, 0.69) were found in the groups with higher Fe contents at HTP and DL townships, indicating these three heavy metals may have an adsorbed form on iron hydroxide in the paddy soils. If human activity’s influence contributed to these positive correlations, e.g., industrial waste disposal, rice grains’ Pb and Cd concentrations should not be at the lowest level. Therefore, we can say that the Ni contents in this study were close to the natural background level. Nevertheless, further geochemical characterisation of the paddy soil is required to discuss whether human industrial activities caused the positive correlations between the heavy metals.

Previous papers [20, 21] reported toxic elemental concentration in rice grains from two townships in the west Ayeyawady delta using ICP-OES, as shown in Figs 4 and 5. The mean values of Pb, Cd, Ni in their reports were out of this study’s ranges, and their data project toward a Pb-Cd enrichment component from the background range, indicating possible pollution. Alternatively, the higher Ni contents in their samples than in this study may indicate the influence of ultramafic rock distribution in Southwest Myanmar [19, 62]. The possibility should be examined by analyzing the rice grains from areas other than this study in the Ayeyawady Delta by ICP-MS.

### Estimation of the potential health risks

**Fig 6:**
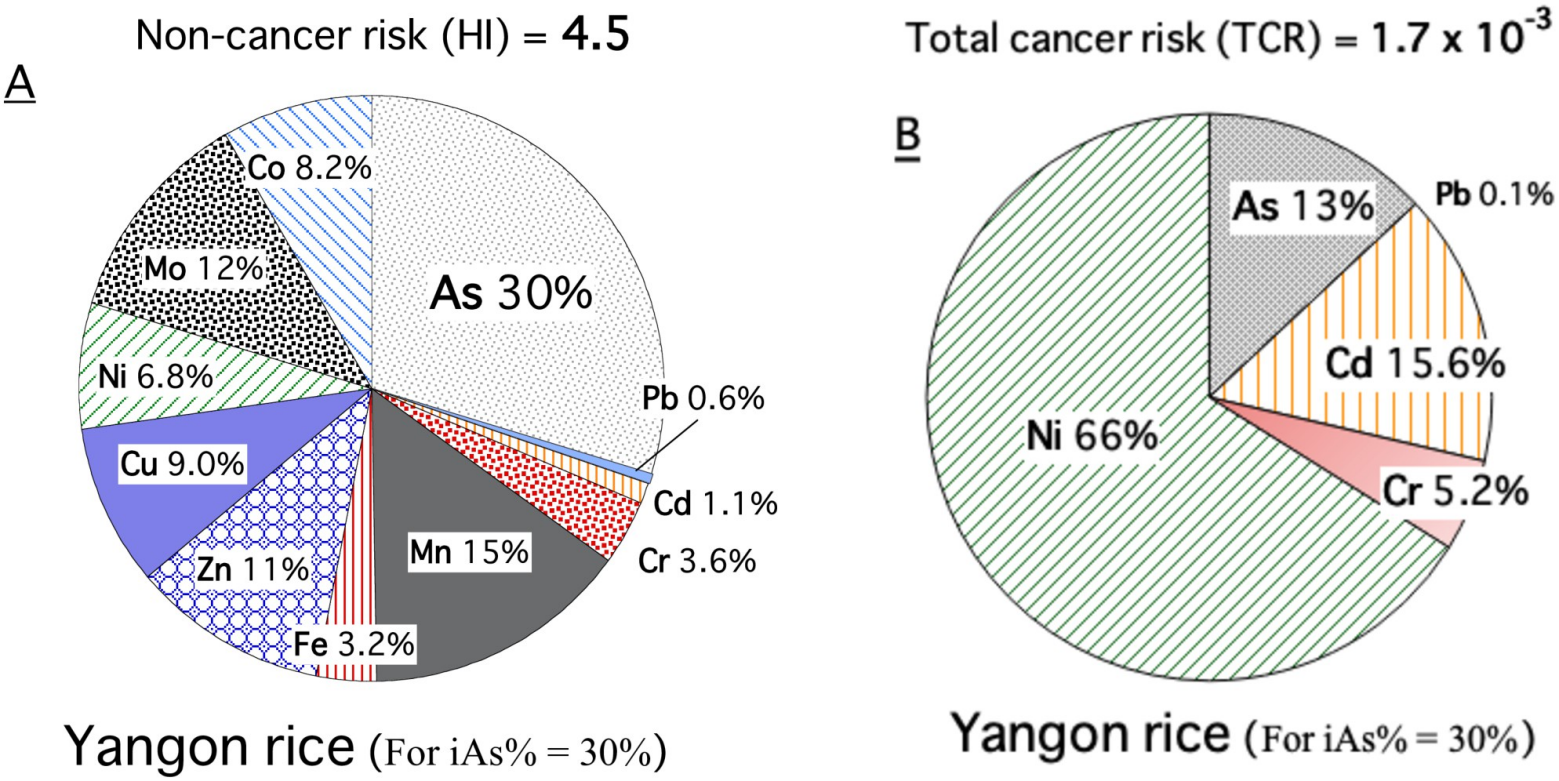
Contribution of individual elements to total health risks for Yangon rice consumers. A) HI represents the sum of each heavy metal’s HQ (non-cancer hazard quotient) values. “HQ = 1” indicates that the level of exposure is likely to cause non-carcinogenic diseases. B) TCR represents total cancer risks, the sum of each heavy metal’s CR (cancer risk) values for exposure duration (26 years). A TCR = 10^−6^ to 10^−4^ indicates that the level of exposure is likely to cause carcinogenic diseases.

Table 1 displays the estimated daily intake (EDI) of each element (μg/kg bw-day), substituting the average daily consumption (0.50 kg rice per day) and the average body weight (58 kg) of Myanmar habitants into the Eq (1). Although we did not analyse the proportion of inorganic As (iAs%) in the Yangon white rice, it is reported that Yangon deltaic rice has 0.04–0.05 mg/kg of inorganic arsenic [14], and the iAs% value was assumed to be about 30% in this paper. Assuming iAs% to be 30%, the total value of the non-cancer hazard quotient index (HI) was 4.5 times the reference value of 1 (Fig 6A). Expressing the value of HI in terms of the iAs(%) equation gives HI = 3.19 + 4.46 × iAs(%)/100. Arsenic (30%) is the main contributor to the HI value, although it changes with iAs%, followed by Mn, Zn, Cu, Mo, Co and Ni (15–7%) and Pb, Cd, Cr and Fe (below 4%).

Assuming that iAs% was 30%, the total lifetime cancer risk (TCR) by these five elements was 1.73 ± 0.59 × 10^−3^, around 17 times higher than the USEPA upper limit of cancer risk for an environmental carcinogen of one case/ten thousand people (Table 1; Fig 6B). TCR = (1.50 + 0.75 × iAs(%)/100)×10^−3^. Nickel (66%) is the main contributor to the cancer risk value, followed by Cd (16%), As (13%), Cr (5.2%) and Pb (0.1%). The Yangon rice grains have average contents of nickel and arsenic worldwide from an unpolluted paddy field, but the lower contents of lead, cadmium and chromium by about one magnitude than the worldwide contents, explaining the high-percentage carcinogenic risks by As and Ni of this study.

The CR value per 10^4^ people for our result was 2.2 (As), 11.4 (Ni), 2.7 (Cd), and 0.012 (Pb) with a TCR of 17.3 per 10^4^ people, and these CR values were calculated with Eq (3) by considering an exposure duration (ED) of 26 years in Yangon, Myanmar. This high value is also contributed by the high rice consumption in the region at 0.5 kg/day. In the case of rural households in West Bengal, India, it was reported that the mean CR value per 10^4^ people for these elements was 5.45 (As), 2.24 (Ni), 6.22 (Cd), and 0.16 (Pb) with a TCR of 14.1 per 10^4^ people with ED of 10 years [1]. As an extension of the ED will increase the value of CR, the comparison suggests that the carcinogenic risk of non-essential trace elements in Yangon rice is around an ordinary level. TCR values from rice consumption at four local sites in Thailand were reported as 3–5 per 10^4^ people by probabilistic risk assessment, which was also higher than the acceptable risk of 1 per 10^4^ people [10, 27].

### Conclusions and future study perspectives

Our study revealed that “Yangon rice” samples from the east end area of the Ayeyawady delta have concentrations comparable to the lowest levels of lead, cadmium, and chromium contents of the literature values and a worldwide average level of As and Ni for the first time. We considered that these contents were natural background levels of the region. The data make it possible to detect and diagnose anthropogenic pollution in other delta parts and future pollution in this area. However, the chemical analysis of phosphorus and these elements in paddy soils and irrigation water is needed to confirm that the results of this study are at the natural background level. If the genotype of rice is responsible for the lowest levels of Pb, Cd and Cr in the world, it will be necessary to preserve the genotype in this region to reduce heavy metal intake. Determining the situation in most other parts of the Ayeyarwady Delta is also necessary. Although arsenic and nickel, but not cadmium, are the main problems due to low cadmium concentrations in Yangon rice grains, cadmium contamination is a problem in other parts of this delta [78].

The total non-cancer hazard quotient index (HI) caused by the Yangon rice’s heavy metals intake was calculated to be 4.5 times the reference value. The As intake accounted for 30%, assuming that iAs% was 30%. The average daily intake of total arsenic in Myanmar is calculated to be 78 ± 26 (μg/person/day) in Table 1. Cardiovascular disease (CVD) in association with iAs exposure are well documented; for example, it is reported that CVD risks increased with iAs exposure from rice at exposures above 0.3 μg/person/day [79]. The potential health hazards of As intake via drinking water in Myanmar have been reported [80, 81]. Therefore, inorganic arsenic intake from rice [14] should also be noted as a potential issue. Various countermeasures (e.g., [12, 18]), such as rice cooking improvement (e.g., [82]) and copper oxide nanoparticles input [41], have been proposed to reduce the As intake amount by rice consumption.

The cancer risk index (TCR) from heavy metal intake in Yangon rice was calculated to be 17 times higher than the USEPA limit, with Ni intake accounting for two-thirds of the index. The high cancer risk of Ni intake has been reported in environmentally contaminated or mafic/ultramafic areas: nickel concentrations in rice in the literature [18, 32, 83] were 0.84 ± 0.40 mg/kg, 2.66 ± 1.46 mg/kg, and 1.22–9.34 mg/kg, which were two times to one order of magnitude higher than the Ni level (0.39 ± 0.19 mg/kg) of this study. Even though the Ni level in Yangon rice grains is around the global average range in uncontaminated and non-mafic areas, the carcinogenic risk of Ni intake from Yangon rice consumption was higher than that of arsenic or cadmium. Although the risk of nickel intake has received less attention, hidden by the magnitude of the risk of Cd and As so far, this study indicates that Ni intake from rice should be highlighted as an alternative potential risk.

Ultramafic rock areas are located in northern Myanmar, and nickel content and estimated daily intake (EDI) in other foods, such as beans [84], remain to be investigated. Unlike arsenic, improving cooking may not reduce nickel intake because Ni and Cd even distribute throughout white rice’s endosperm, while As is localised in the outer surface of a white rice grain [85]. The geochemical behaviour of nickel in paddy soils [86] allows envisaging the improvement to reduce the bioaccumulation of nickel in rice grains remains challenged. The rice genotype might be a helpful tool for mitigating Ni toxicity [22].

## Supporting information

S1 Table Locations of sampling sites from five townships of the Yangon region in Myanmar. (PDF)

S2 Table The comparison between analytical results and certified literature values (mg/kg) for four certified reference materials (CRMs), with mean and standard deviation (SD) values in repeated digestion number (n). NMIJ: National Metrology Institute of Japan; NIES: National Institute of Environmental Studies, Japan Environmental Agency: CRM: Certified Reference Material. *: NMIJ CRM 7501-a, Trace elements in White Rice Flour (Cd level I), 2017; https://unit.aist.go.jp/nmij/english/refmate/crm/cert/7501a_en.pdf **: NMIJ CRM 7502-a, Trace elements in White Rice Flour (Cd level II), 2017; https://unit.aist.go.jp/nmij/english/refmate/crm/cert/7502a_en.pdf. $: NIES CRM No.10, Low-Cd; Rice Flour-Unpolished (Brown rice), 1989; http://www.speciation.net/Database/Materials/National-Institute-for-Environmental-Studies-NIES/NIES-CRM-10-a-Rice-FlourUnpolished-Low-Level-Cadmium-;i371. #: NIES CRM No.9, Sargasso, seaweed, 1988; https://unit.aist.go.jp/nmij/english/refmate/rminfo/index.html. &: The number in parentheses is the reference value; &$: Contamination from the rotary mill during sample crushing.
(PDF)

S3 Table Concentrations (mg/kg) of 11 elements in 120 white rice samples from five townships (TK, HTP, DL, KM, KCK) in Myanmar. *LOQ: limitations of quantitation (= standard deviation (SD) x 10) of blank solutions were converted into rice sample abundance (mg/kg). Values below LOQ and above LOD (limitation of detection) are shown in parentheses, and the inequality sign shows values below LOD. The accuracy of the data was confirmed by replicating the further sample analysis, and the value that could not be confirmed was shown as "-----".
(PDF)

S4 Table Concentrations (mg/kg) of 11 elements in 120 white rice samples from five townships (TK, HTP, DL, KM, KCK) in Myanmar. Values that the absolute value of the correlation coefficient exceeds 0.65 are surrounded by a square.
(PDF)
