# Supplementary material for: Arsenic and heavy metal contents in white rice samples from rainfed paddy fields in Yangon division, Myanmar—Natural background levels?
Source: PLoS One. 2023 Mar 24;18(3):e0283420. doi: 10.1371/journal.pone.0283420 (PMC10038304; doi:10.1371/journal.pone.0283420)
Supplement: S1 Table — (PDF) [file pone.0283420.s001.pdf]

S1 Table. Locations of sampling sites from five townships of Yangon region in Myanmar.

Township name	irrigation method	Village Code	Sample No.	Altitude (m)	Latitude	Longitude
Taik Kyi (**TK**)	Rainfed, Irrigation	TK 1	1–5	15.5	17°18'01.86"N	95°57'15.14"E
		TK 2	6–10	12.5	17°16'32.61"N	95°56'35.42"E
		TK 3	11–15	11.0	17°16'15.39"N	95°56'48.77"E
		TK 4	16–19	18.0	17°17'02.88"N	95°56'38.22"E
		TK 5	21–24	16.0	17°16'36.62"N	95°56'23.53"E
Htan Ta Pin (**HTP**)	Rainfed	HTP 1	1–5	11.0	17°20'40.43"N	95°52'32.58"E
		HTP 2	6–10	6.0	17°12'48.46"N	95°56'32.52"E
		HTP 3	11–15	13.0	17° 09'45.19"N	95°52'47.88"E
		HTP 4	16–20	12.0	17°10'19.98"N	95°52'07.94"E
		HTP 5	21–25	13.0	17°10'38.33"N	95°51'29.49"E
Dala (**DL**)	Rainfed	DL 1	1–5	6.0	16°37'52.99"N	96° 06'32.05"E
		DL 2	6–10	8.0	16°40'34.32"N	96° 08'50.59"E
		DL 3	11–15	5.0	16°40'13.90"N	96° 08'11.85"E
		DL 4	16–20	5.5	16°37'51.42"N	96° 08'40.26"E
		DL 5	21–24	4.0	16°43'22.36"N	96° 08'16.85"E
Kaw Mhu (**KM**)	Rainfed	KM 1	1–5	7.0	16°37'07.40"N	96° 05'23.87"E
		KM 2	6–10	4.0	16°31'15.92"N	96° 10'0.62"E
		KM 3	11–15	6.0	16°28'12.83"N	96° 08'53.47"E
		KM 4	16–20	5.0	16°36'43.86"N	96° 05'28.71"E
		KM 5	21–25	6.0	16°29'24.56"N	96° 09'20.10"E
Kon Chan Kone (**KCK**)	Rainfed	KCK 1	1–5	9.0	16°25'40.10"N	96° 06'13.33"E
		KCK 2	6–10	11.0	16°25'23.45"N	96° 05'40.40"E
		KCK 3	11–15	6.0	16°26'06.24"N	96° 09'38.92"E
		KCK 4	16–20	3.0	16°27'10.35"N	96° 07'57.74"E
		KCK 5	21, 22	9.0	16°26'37.35"N	96° 11'5.41"E
