# Supplementary material for: Arsenic and heavy metal contents in white rice samples from rainfed paddy fields in Yangon division, Myanmar—Natural background levels?
Source: PLoS One. 2023 Mar 24;18(3):e0283420. doi: 10.1371/journal.pone.0283420 (PMC10038304; doi:10.1371/journal.pone.0283420)
Supplement: S2 Table — NMIJ: National Metrology Institute of Japan; NIES: National Institute of Environmental Studies, Japan Environmental Agency: CRM: Certified Reference Material. *: NMIJ CRM 7501-a, Trace elements in White Rice Flour (Cd level I), 2017; https://unit.aist.go.jp/nmij/english/refmate/crm/cert/7501a_en.pdf **: NMIJ CRM 7502-a, Trace elements in White Rice Flour (Cd level II), 2017; https://unit.aist.go.jp/nmij/english/refmate/crm/cert/7502a_en.pdf. $: NIES CRM No.10, Low-Cd; Rice Flour-Unpolished (Brown rice), 1989; http://www.speciation.net/Database/Materials/National-Institute-for-Environmental-Studies-NIES/NIES-CRM-10-a-Rice-FlourUnpolished-Low-Level-Cadmium-;i371. #: NIES CRM No.9, Sargasso, seaweed, 1988; https://unit.aist.go.jp/nmij/english/refmate/rminfo/index.html. &: The number in parentheses is the reference value; &$: Contamination from the rotary mill during sample crushing. (PDF) [file pone.0283420.s002.pdf]

**S2 Table.** The comparison between analytical results and certified literature values (mg/kg) for four certified reference materials (CRMs), with mean and standard deviation (SD) values in repeated digestion number (n).

| Mean ±1SD | NMIJ CRM 7501-a* | | NMIJ CRM 7502-a** | | NIES CRM No.10, low-Cd $ | | NIES CRM No.9 Sargasso # | |
|---|---|---|---|---|---|---|---|---|
| | **Results (n = 18)** | **Certified values&** | **Results (n = 14)** | **Certified values&** | **Results (n = 8)** | **Certified values&** | **Results (n = 10)** | **Certified values&** |
| **As** | 0.122 ± 0.002 | | 0.120 ± 0.010 | 0.109 ± 0.005 | 0.213 ± 0.011 | (0.17) | 109 ± 4 | 115 ± 9 |
| **Pb** | 0.0028 ± 0.0013 | | 0.0051 ± 0.0012 | 0.0043 ± 0.0006 | 0.979 ± 0.053 | (1)&$ | 1.38 ± 0.15 | 1.35 ± 0.05 |
| **Cd** | 0.0521 ± 0.0009 | 0.0517 ± 0.0024 | 0.520 ± 0.030 | 0.548 ± 0.020 | 0.0254 ± 0.0015 | 0.023 ± 0.003 | 0.181 ± 0.019 | 0.15 ± 0.02 |
| **Cr** | 0.116 ± 0.020 | | 0.078 ± 0.023 | 0.075 ± 0.013 | 0.068 ± 0.028 | (0.07) | | (0.2) |
| **Mn** | 6.65 ± 0.18 | 6.75 ± 0.26 | 10.4 ± 0.7 | 11.2 ± 0.4 | 32.6 ± 2.1 | 34.7 ± 1.8 | 23.6 ± 1.3 | 21.2 ± 1.0 |
| **Fe** | 3.91 ± 0.22 | 4.04 ± 0.24 | 4.63 ± 0.88 | 4.48 ± 0.20 | 11.36 ± 0.77 | 12.7 ± 0.7 | 187 ± 14 | 187 ± 6 |
| **Zn** | 20.1 ± 0.3 | 20.1 ± 0.7 | 24.1 ± 1.6 | 26.0 ± 0.9 | 23.7 ± 1.5 | 25.2 ± 0.8 | 16.4 ± 1.6 | 15.6 ± 1.2 |
| **Cu** | 2.52 ± 0.05 | 2.49 ± 0.09 | 2.82 ± 0.18 | 3.02 ± 0.11 | 3.46 ± 0.19 | 3.5 ± 0.3 | 4.9 ± 0.4 | 4.9 ± 0.2 |
| **Ni** | 0.192 ± 0.010 | | 0.36 ± 0.03 | 0.39 ± 0.02 | 0.146 ± 0.036 | 0.19 ± 0.03 | 67.9 ± 6.5 | |
| **Mo** | 0.524 ± 0.009 | 0.556 ± 0.022 | 0.80 ± 0.05 | 0.79 ± 0.03 | 0.334 ± 0.017 | 0.35 ± 0.05 | | |
| **Co** | 0.017 ± 0.004 | | 0.010 ± 0.004 | | 0.022 ± 0.011 | (0.02) | | 0.12 ± 0.01 |

NMIJ: National Metrology Institute of Japan; NIES: National Institute of Environmental Studies, Japan Environmental Agency: CRM: Certified Reference Material

*: NMIJ CRM 7501-a, Trace elements in White Rice Flour (Cd level I), 2017; https://unit.aist.go.jp/nmij/english/refmate/crm/cert/7501a_en.pdf

**: NMIJ CRM 7502-a, Trace elements in White Rice Flour (Cd level II), 2017; https://unit.aist.go.jp/nmij/english/refmate/crm/cert/7502a_en.pdf

$: NIES CRM No.10, Low-Cd; Rice Flour-Unpolished (Brown rice), 1989; http://www.speciation.net/Database/Materials/National-Institute-for-Environmental-Studies-NIES/NIES-CRM-10-a-Rice-FlourUnpolished-Low-Level-Cadmium-;i371

#: NIES CRM No.9, Sargasso, seaweed, 1988; https://unit.aist.go.jp/nmij/english/refmate/rminfo/index.html

&: The number in parentheses is the reference value; &$: Contamination from the rotary mill during sample crushing
