# Supplementary material for: Arsenic and heavy metal contents in white rice samples from rainfed paddy fields in Yangon division, Myanmar—Natural background levels?
Source: PLoS One. 2023 Mar 24;18(3):e0283420. doi: 10.1371/journal.pone.0283420 (PMC10038304; doi:10.1371/journal.pone.0283420)
Supplement: S3 Table — *LOQ: limitations of quantitation (= standard deviation (SD) x 10) of blank solutions were converted into rice sample abundance (mg/kg). Values below LOQ and above LOD (limitation of detection) are shown in parentheses, and the inequality sign shows values below LOD. The accuracy of the data was confirmed by replicating the further sample analysis, and the value that could not be confirmed was shown as "-----". (PDF) [file pone.0283420.s003.pdf]

S3 Table. Concentrations (mg/kg) of 11 elements in 120 white rice samples from five townships (TK, HTP, DL, KM, KCK) in Myanmar.

Area name		As	Pb	Cd	Cr	Mn	Fe	Zn	Cu	Ni	Mo	Co
	**No.**	120	115	115	118	120	119	117	119	119	120	119
**TK**	**1**	0.240	0.0076	0.0024	0.051	10.2	5.0	19.2	0.74	0.273	0.251	0.0194
	**2**	0.120	0.0060	0.0074	0.075	13.7	4.7	17.8	1.48	0.570	0.221	0.0153
	**3**	0.083	0.0093	------	0.087	11.1	5.3	11.9	1.87	0.563	0.361	0.0066
	**4**	0.180	0.0374	0.0017	0.065	12.3	4.6	19.5	1.35	0.317	0.135	0.0156
	**5**	0.174	0.0075	0.0049	0.107	12.1	6.5	17.3	1.72	0.484	0.228	0.0149
	**6**	0.234	0.0034	0.0030	0.102	10.4	4.0	19.2	0.74	0.253	0.247	0.0187
	**7**	0.143	------	0.0030	0.080	20.9	13.4	19.2	1.88	0.502	0.177	0.0260
	**8**	0.154	0.0054	0.0033	0.059	11.4	4.0	13.5	1.25	0.277	0.116	0.0101
	**9**	0.185	0.0150	0.0022	0.096	12.2	9.8	20.2	1.39	0.341	0.146	0.0174
	**10**	0.250	0.0039	0.0022	0.068	11.1	3.2	20.3	0.75	0.270	0.247	0.0178
	**11**	0.105	0.0056	0.0070	0.066	13.3	4.2	16.7	1.40	0.555	0.211	0.0126
	**12**	0.149	0.0040	0.0026	0.058	11.4	3.3	13.7	1.25	0.273	0.121	0.0091
	**13**	0.142	0.0152	0.0050	0.070	12.4	4.3	13.2	1.26	0.299	0.235	0.0098
	**14**	0.103	0.0165	0.0061	0.140	8.9	5.7	20.1	1.47	0.367	0.307	0.0148
	**15**	0.290	0.0052	0.0033	0.126	14.1	5.2	23.8	0.93	0.356	0.309	0.0215
	**16**	0.151	0.0061	0.0027	0.057	11.6	3.0	13.7	1.21	0.270	0.130	0.0090
	**17**	0.108	0.0042	0.0043	0.093	13.3	5.0	16.8	1.36	0.508	0.208	0.0126
	**18**	0.203	0.0235	0.0019	0.071	13.0	5.8	22.5	1.56	0.366	0.161	0.0182
	**19**	0.116	0.0071	0.0072	0.052	13.6	17.4	18.0	1.51	0.552	0.226	0.0120
	**20**	0.157	0.0092	0.0044	0.080	9.7	6.3	15.1	1.75	0.199	0.398	0.0091
	**21**	0.115	0.0071	0.0063	0.043	12.9	4.7	17.5	1.47	0.537	0.219	0.0105
	**22**	0.263	0.0061	0.0025	0.100	10.7	3.8	20.1	0.78	0.259	0.262	0.0180
	**23**	0.084	0.0161	------	0.058	16.2	4.0	12.7	2.35	0.887	0.197	0.0077
	**24**	0.189	0.0067	0.0031	0.061	9.2	5.0	22.4	1.27	0.177	0.340	0.0124
**HTP**	**1**	0.133	0.0137	0.0028	0.101	6.9	22.3	18.1	1.49	0.182	0.236	0.0172
	**2**	0.097	0.0037	------	0.051	8.7	3.4	20.5	2.36	0.289	0.658	0.0038
	**3**	0.124	0.0075	0.0047	0.055	9.3	4.6	13.4	1.76	0.174	0.338	0.0083
	**4**	0.133	0.0117	0.0045	0.068	10.4	6.2	14.7	1.73	0.277	0.311	0.0083
	**5**	0.131	0.0071	0.0036	0.095	9.9	35.8	18.7	1.47	0.402	0.442	0.0225
	**6**	0.110	0.0036	0.0097	0.065	11.6	6.3	12.3	3.58	1.055	0.222	0.0122

	**7**	0.199	0.0071	0.0038	0.069	11.2	5.8	20.6	2.09	0.183	0.492	0.0118
	**8**	0.133	0.0141	0.0027	0.052	7.0	39.9	17.7	1.36	0.175	0.203	0.0180
	**9**	0.113	0.0069	0.0033	0.064	10.1	6.8	19.5	2.28	0.362	0.322	0.0090
	**10**	0.086	0.0283	0.0052	0.087	9.7	35.6	11.9	2.48	0.638	0.256	0.0277
	**11**	0.099	0.0124	0.0072	0.033	8.4	6.3	16.4	2.05	0.447	0.333	0.0079
	**12**	0.145	0.0119	0.0036	0.047	10.6	5.8	14.9	1.85	0.247	0.357	0.0099
	**13**	0.132	0.0087	0.0092	0.056	7.8	15.8	16.5	2.03	0.444	0.482	0.0093
	**14**	0.176	0.0145	0.0021	0.033	12.2	4.7	19.4	1.27	0.307	0.141	0.0150
	**15**	0.142	0.0116	0.0087	0.036	10.8	8.5	21.3	2.48	0.541	0.493	0.0129
	**16**	0.126	0.0130	0.0039	0.045	9.5	43.7	14.5	1.74	0.231	0.314	0.0099
	**17**	0.133	0.0077	0.0027	0.055	8.8	52.6	17.1	1.37	0.390	0.458	0.0193
	**18**	0.140	0.0147	0.0015	0.079	7.0	41.2	18.1	1.38	0.193	0.207	0.0193
	**19**	0.136	0.0094	0.0074	0.029	10.5	19.3	12.2	2.44	0.513	0.403	0.0121
	**20**	0.047	0.0123	------	0.082	12.9	16.4	------	------	------	0.501	0.0286
	**21**	0.094	0.0141	0.0050	0.037	8.3	16.9	19.6	1.32	0.198	0.268	0.0098
	**22**	0.109	0.0120	0.0063	0.047	8.9	32.5	19.2	2.29	0.540	0.398	0.0084
	**23**	0.135	0.0068	0.0075	0.109	7.2	------	18.9	1.89	0.434	0.490	0.0123
	**24**	0.152	0.0105	0.0099	0.041	11.6	8.2	13.5	2.74	0.588	0.474	0.0142
	**25**	0.147	0.0248	0.0057	0.058	17.3	25.9	15.3	2.42	0.485	0.311	0.0117
**DL**	**1**	0.107	0.0073	0.0047	0.056	8.9	11.1	18.2	2.13	0.326	0.298	0.0096
	**2**	0.118	(0.0018)	0.0035	0.044	8.7	7.0	17.6	1.96	0.430	0.237	------
	**3**	0.194	0.0028	0.0016	0.037	16.2	14.8	24.1	1.47	0.406	0.310	0.0300
	**4**	0.238	0.0371	0.0140	0.062	13.9	7.6	14.8	2.85	0.625	0.344	0.0121
	**5**	0.100	(0.0021)	0.0100	0.021	9.0	4.9	11.6	3.29	0.935	0.190	0.0108
	**6**	0.148	0.0066	0.0069	0.031	10.0	12.4	17.6	1.64	0.321	0.345	0.0098
	**7**	0.164	0.0035	0.0051	0.024	7.7	25.6	17.0	2.14	0.289	0.332	0.0096
	**8**	0.163	(0.0019)	0.0022	0.021	7.0	6.9	22.8	1.41	0.260	0.421	0.0108
	**9**	0.216	0.0307	0.0129	0.026	12.3	5.9	------	2.53	0.543	0.331	0.0242
	**10**	0.129	0.0087	0.0011	(0.014)	7.7	9.9	18.3	1.75	0.168	0.270	0.0077
	**11**	0.208	0.0153	0.0024	0.022	17.1	19.2	19.8	2.17	0.441	0.365	0.0146
	**12**	0.177	0.0225	0.0132	(0.015)	17.8	9.0	19.9	2.27	0.534	0.246	0.0127
	**13**	0.223	0.0182	0.0019	0.026	22.0	15.7	20.3	2.15	0.461	0.485	0.0105
	**14**	0.317	0.0294	0.0022	0.073	19.4	21.0	22.2	2.34	0.581	0.554	0.0242
	**15**	0.160	0.0068	0.0017	0.020	8.8	18.7	------	1.26	0.203	0.271	0.0243

	**16**	0.196	0.0368	0.0124	0.090	11.4	8.1	13.1	2.52	0.510	0.300	0.0088
	**17**	0.115	------	0.0168	0.051	18.5	14.5	14.3	3.02	0.867	0.395	0.0098
	**18**	0.190	0.0082	0.0012	0.020	15.1	13.1	18.6	1.69	0.223	0.373	0.0154
	**19**	0.194	0.0247	0.0054	0.035	15.2	18.7	18.0	2.36	0.532	0.295	0.0181
	**20**	0.299	0.0202	0.0043	0.024	21.9	15.6	22.5	2.28	0.456	0.607	0.0152
	**21**	0.154	0.0136	0.0012	0.018	13.4	9.4	18.6	1.92	0.227	0.262	0.0108
	**22**	0.348	0.0317	0.0016	(0.017)	20.6	35.8	22.4	2.37	0.459	0.584	0.0185
	**23**	0.216	0.0215	0.0126	(0.017)	13.1	11.3	14.1	2.21	0.522	0.327	0.0155
	**24**	0.124	0.0141	0.0055	------	10.1	15.4	18.2	1.48	0.467	0.208	0.0190
**KM**	**1**	0.206	0.0082	0.0034	0.045	11.3	13.5	15.3	2.73	0.710	0.274	0.0205
	**2**	0.225	(0.0013)	0.0102	0.078	8.3	2.1	15.7	1.43	0.191	0.389	0.0059
	**3**	0.156	0.0056	0.0051	0.092	10.4	7.5	20.2	2.22	0.642	0.209	0.0116
	**4**	0.159	0.0047	0.0058	0.090	11.0	7.2	19.1	1.87	0.381	0.285	0.0096
	**5**	0.244	0.0084	0.0041	0.075	13.0	10.2	18.8	1.80	0.254	0.498	0.0178
	**6**	0.230	(0.0016)	0.0100	0.104	8.0	2.0	15.6	1.46	0.206	0.414	0.0076
	**7**	0.100	------	0.0114	0.062	7.2	3.5	13.6	1.88	0.418	0.275	0.0114
	**8**	0.138	(0.0017)	0.0019	0.067	9.5	4.8	20.5	1.44	0.188	0.249	0.0093
	**9**	0.150	0.0050	0.0036	0.113	9.5	8.9	19.0	2.36	0.425	0.394	0.0145
	**10**	0.098	0.0093	------	0.103	13.0	3.4	13.8	2.06	0.589	0.401	0.0073
	**11**	0.176	0.0032	0.0086	0.063	8.3	4.4	14.3	1.70	0.212	0.332	0.0056
	**12**	0.136	0.0064	0.0029	0.072	8.6	5.5	19.1	2.33	0.225	0.254	0.0091
	**13**	0.109	0.0231	0.0123	0.067	7.4	3.4	14.6	1.97	0.410	0.320	0.0117
	**14**	0.119	------	0.0025	0.074	8.0	3.8	17.3	1.32	0.174	0.241	0.0079
	**15**	0.217	< 0.0007	0.0083	0.138	7.7	2.0	14.6	1.38	0.182	0.388	0.0067
	**16**	0.153	0.0031	0.0050	0.097	9.8	7.0	19.6	1.84	0.556	0.143	0.0111
	**17**	0.110	0.0105	0.0108	0.037	11.4	5.0	12.3	3.39	1.057	0.207	0.0136
	**18**	0.196	0.0067	0.0026	0.040	8.5	5.1	18.5	1.98	0.125	0.452	0.0107
	**19**	0.152	0.0070	0.0052	0.052	8.3	5.7	16.7	2.29	0.428	0.272	0.0090
	**20**	0.151	0.0045	0.0062	0.093	12.8	4.4	16.6	1.12	0.337	0.233	0.0157
	**21**	0.128	0.0083	< 0.0003	0.045	8.7	8.4	21.4	2.05	0.318	0.265	0.0093
	**22**	0.107	0.0067	0.0097	0.026	7.4	4.7	13.1	1.27	0.197	0.507	0.0112
	**23**	0.175	0.0069	0.0026	(0.006)	8.7	12.0	17.5	1.17	0.157	0.424	0.0101
	**24**	0.111	0.0033	0.0123	0.066	7.0	6.0	13.0	1.36	0.223	0.587	0.0146
	**25**	0.153	0.0104	(0.0007)	0.065	19.6	16.8	22.1	2.24	0.243	0.119	0.0210

**KCK**	**1**	0.194	0.0046	0.0053	------	23.0	13.8	24.2	2.08	0.434	0.278	0.0237
	**2**	0.143	(0.0022)	0.0028	0.027	7.2	4.2	18.9	1.74	0.210	0.319	0.0100
	**3**	0.137	(0.0018)	0.0116	0.034	7.1	3.9	14.9	1.67	0.248	0.162	0.0035
	**4**	0.086	0.0029	0.0089	(0.016)	8.0	11.8	12.9	3.05	0.609	0.271	0.0119
	**5**	0.134	0.0051	0.0049	(0.017)	7.5	19.3	18.1	1.47	0.171	0.233	0.0096
	**6**	0.087	(0.0010)	0.0091	0.022	7.8	4.1	11.8	3.00	0.571	0.262	0.0134
	**7**	0.142	0.0023	0.0139	0.063	7.5	4.0	14.9	1.74	0.259	0.169	0.0049
	**8**	0.180	0.0033	0.0030	0.036	7.5	7.8	16.1	1.55	0.295	0.338	0.0097
	**9**	0.192	------	(0.0010)	0.050	13.1	31.2	18.2	2.07	0.256	0.296	0.0213
	**10**	0.136	0.0097	0.0091	< 0.006	10.8	10.2	21.1	2.35	0.577	0.465	0.0119
	**11**	0.140	0.0030	0.0052	< 0.006	9.2	5.6	19.4	1.92	0.266	0.408	0.0135
	**12**	0.082	0.0042	0.0084	0.055	7.3	4.4	11.3	2.98	0.600	0.258	0.0122
	**13**	0.194	0.0053	0.0037	(0.008)	7.1	3.6	14.2	1.52	0.137	0.473	0.0050
	**14**	0.189	0.0047	0.0033	0.052	9.2	5.9	18.4	1.71	0.352	0.346	0.0108
	**15**	0.081	0.0029	0.0078	0.067	7.6	42.5	11.0	2.79	0.578	0.237	0.0120
	**16**	0.142	(0.0017)	0.0053	0.029	7.0	3.1	13.5	1.27	0.183	0.219	0.0061
	**17**	0.159	0.0032	0.0047	0.048	7.9	4.1	18.6	1.85	0.245	0.332	0.0075
	**18**	0.198	0.0058	0.0028	0.067	8.5	6.5	17.9	1.73	0.314	0.339	0.0117
	**19**	0.079	(0.0011)	0.0092	0.037	7.6	2.7	10.9	2.99	0.617	0.329	0.0108
	**20**	0.139	0.0090	0.0095	0.052	7.7	9.6	20.9	2.03	0.451	0.511	0.0132
	**21**	0.113	0.0085	0.0161	0.030	11.7	6.3	21.5	2.37	0.729	0.356	0.0115
	**22**	0.185	0.0034	0.0031	0.027	7.7	4.3	16.3	1.53	0.275	0.323	0.0089
**LOQ***		0.0016	0.0022	0.00103	0.018	0.013	0.625	0.072	0.0095	0.025	0.033	0.0025

*LOQ: limitations of quantitation (= standard deviation (SD) x 10) of blank solutions were converted into rice sample abundance (mg/kg).

Values below LOQ and above LOD (limitation of detection) are shown in parentheses, and the inequality sign shows values below LOD.

The accuracy of the data was confirmed by replicating the further sample analysis, and the value that could not be confirmed was shown as "-----".
