# Supplementary material for: Arsenic and heavy metal contents in white rice samples from rainfed paddy fields in Yangon division, Myanmar—Natural background levels?
Source: PLoS One. 2023 Mar 24;18(3):e0283420. doi: 10.1371/journal.pone.0283420 (PMC10038304; doi:10.1371/journal.pone.0283420)
Supplement: S4 Table — Values that the absolute value of the correlation coefficient exceeds 0.65 are surrounded by a square. (PDF) [file pone.0283420.s004.pdf]

S4 Table. The correlation coefficient table between ten elemental contents in sample grains by five townships (TK, HTP, DL, KM, KCK). Values that the absolute value of the correlation coefficient exceeds 0.65 are surrounded by a square.

**TK**	Mn	Fe	Co	**Ni**	Cu	Zn	As	Mo	Cd	Pb
Mn	1									
Fe	0.45	1								
Co	0.34	0.28	1							
Ni	0.61	0.23	-0.20	1						
**Cu**	0.47	0.31	-0.38	0.70	1					
Zn	-0.03	0.19	0.78	-0.31	-0.43	1				
As	-0.23	-0.18	0.63	-0.63	-0.75	0.65	1			
Mo	-0.37	-0.01	-0.07	-0.11	0.00	0.15	0.10	1		
**Cd**	0.15	-0.05	-0.50	0.69	0.61	-0.54	-0.54	0.28	1	
Pb	0.40	0.26	0.28	0.11	0.39	0.17	-0.10	-0.27	0.00	1

**HTP**	Mn	Fe	Co	**Ni**	Cu	Zn	As	Mo	Cd	Pb
Mn	1									
Fe	-0.35	1								
Co	0.07	0.24	1							
Ni	0.42	-0.05	0.46	1						
**Cu**	0.50	-0.24	0.11	0.83	1					
Zn	-0.03	0.05	0.18	0.23	0.02	1				
As	0.13	-0.00	-0.24	-0.57	-0.42	-0.17	1			
Mo	0.03	0.08	-0.16	0.21	0.33	0.35	-0.09	1		
**Cd**	0.31	-0.08	0.44	0.73	0.76	0.50	-0.60	0.39	1	
Pb	0.28	0.04	0.41	0.01	-0.08	-0.20	-0.10	-0.47	-0.03	1

**DL**	Mn	Fe	Co	**Ni**	Cu	Zn	As	Mo	Cd	Pb
Mn	1									
Fe	0.42	1								
Co	-0.10	-0.08	1							
Ni	0.32	-0.14	-0.02	1						
**Cu**	0.30	-0.10	-0.17	0.84	1					
Zn	-0.02	0.16	0.18	-0.36	-0.43	1				
As	0.70	0.55	-0.05	0.05	0.14	0.19	1			
Mo	0.69	0.54	-0.14	0.01	0.10	0.10	0.82	1		
**Cd**	0.01	-0.40	-0.18	0.69	0.66	-0.27	-0.14	-0.23	1	
Pb	0.44	0.09	-0.19	0.58	0.56	-0.16	0.11	0.25	0.61	1

**KM**	Mn	Fe	Co	**Ni**	Cu	Zn	As	Mo	Cd	Pb
Mn	1									
Fe	0.64	1								
Co	0.63	0.74	1							
Ni	0.25	0.12	0.30	1						
**Cu**	0.32	0.32	0.35	0.78	1					
Zn	0.40	0.52	0.18	-0.22	0.04	1				
As	0.08	0.13	0.02	-0.29	-0.16	0.19	1			
Mo	-0.42	-0.22	-0.13	-0.42	-0.41	-0.44	0.22	1		
**Cd**	0.02	-0.43	-0.27	0.27	0.01	-0.60	-0.31	0.29	1	
Pb	-0.13	-0.12	-0.05	-0.04	-0.07	-0.09	-0.37	-0.21	-0.04	1

**KCK**	Mn	Fe	Co	**Ni**	Cu	Zn	As	Mo	Cd	Pb
Mn	1									
Fe	0.24	1								
Co	0.78	0.46	1							
Ni	0.18	0.13	0.34	1						
**Cu**	0.07	0.22	0.37	0.88	1					
Zn	0.62	-0.02	0.41	-0.14	-0.40	1				
As	0.33	-0.09	0.10	-0.68	-0.77	0.56	1			
Mo	0.04	-0.16	0.16	0.09	-0.04	0.44	0.26	1		
**Cd**	-0.06	-0.16	-0.23	0.59	0.43	-0.12	-0.63	-0.19	1	
Pb	0.35	0.47	0.53	-0.02	-0.03	0.39	0.34	0.28	-0.21	1
